# Prognostic and Predictive Value of an Immunoscore Signature in Glioblastoma Multiform

**DOI:** 10.3389/fgene.2020.514363

**Published:** 2020-11-09

**Authors:** Xiangjun Tang, Pengfei Xu, Ann Chen, Gang Deng, Shenqi Zhang, Lun Gao, Longjun Dai, Qianxue Chen

**Affiliations:** ^1^Department of Neurosurgery, Renmin Hospital of Wuhan University, Wuhan, China; ^2^Department of Neurosurgery, Taihe Hospital, Hubei University of Medicine, Shiyan, China; ^3^The Seventh Affiliated Hospital of Sun Yat-sun University, Guangzhou, China; ^4^Department of Biomedical Engineering, Yale University, New Haven, CT, United States

**Keywords:** glioblastoma multiform, immunoscore, predictive, prognostic, chemotherapy

## Abstract

**Background:**

Although increasing evidence shows that immune infiltration plays an essential role in glioblastoma (GBM), current prognostic indicators do not accurately represent the risk of immune cells infiltration in patients. It is therefore critical to identify new prognostic markers for GBM. Here, we investigated the effectiveness of using immunoscore to improve risk stratification and prediction of prognosis in GBM patients receiving chemotherapy.

**Methods:**

Using mRNA microarrays and CIBERSORT, we analyzed 22 types of immune cell fractions in 517 GBM samples and characterized an immunoscore using the least absolute shrinkage and selection operator (LASSO) Cox regression model based on the fraction of immune cell types and patients’ overall survival. The prognostic and predictive accuracy of immunoscore was verified in the validation cohort and the entire cohort.

**Results:**

Using the LASSO model, an immunoscore was developed to classify patients into High and Low immunoscore groups in the training cohort (*P* < 0.0001) based on the fraction of eight immune cell types. The immunoscore performance was validated in the validation cohort (*P* < 0.0001) and the entire cohort (*P* < 0.0001). Furthermore, a nomogram comprising age, IDH1 status, and immunoscore was generated to predict one- and three-year survival rates in the training cohort. The predictive value of the immunoscore was also confirmed in the validation cohort and the entire cohort (C-index: 0.66, 0.67, and 0.68, respectively). In addition, we concluded that patients in the low-immunoscore group may benefit from adjuvant chemotherapy for GBM.

**Conclusion:**

Immunoscore, an immune-infiltration-based signature, is a reliable prognostic and predictive tool for GBM.

## Introduction

Glioblastoma (GBM) is the most common and malignant variant of intrinsic glial brain tumors (Thakkar et al., 2014). The complicated diagnostic process, lack of methods for predicting sensitivity or resistance to chemotherapy, as well as limited availability of optimal prognostic indicators contribute to the poor prognosis of patients with GBM, which has not significantly improved despite the development of innovative diagnostic methods and new therapies. Therefore, elucidation of the molecular mechanism underlying the aggressiveness of GBM, and identification of appropriate prognostic markers and therapeutic targets, may lead to timely diagnosis, effective therapies, and reliable prediction of prognosis. Recently, genomic, epigenomic, transcriptomic, and proteomic studies of GBM have identified promising candidates as predictive and prognostic biomarkers. In particular, epidermal growth factor receptor (EGFR) overexpression (Nicholas et al., 2006; Huang et al., 2009), isocitrate dehydrogenase 1/2 (IDH1/2) mutations (Dang et al., 2009; Brennan et al., 2013), and O6-methylguanine-DNA methyltransferase (MGMT) promoter methylation (Zawlik et al., 2009) are regarded as having high clinical significance. Numerous new targeted therapies and predictive biomarkers are being evaluated in clinical trials for patients with GBM (Wen et al., 2014; Gilbert et al., 2017). However, the clinical prediction of, and treatment outcomes for patients with, GBM remain unsatisfactory. Omics diagnostics may well understand GBM heterogeneity and may be useful in identifying whether patients can undergo a certain treatment, but the heterogeneity of host immune cells was not mentioned and cannot predict the treatment response.

Recent studies have indicated that immune infiltration plays a significant role in glioma development (Berghoff et al., 2017). The immune response is a complex process involving the integrated activities of multiple cell types such as cytotoxic lymphocytes, antigen-presenting cells, and myeloid cells; however, their functions are significantly altered in glioma tumors. Therefore, evaluation of the extent of immune infiltration is necessary to elucidate GBM tumorigenesis and progression. Some latest research revealed that tumors lacking central memory CD4 T cells or natural killer cells were associated with better prognosis in GBM (Wu et al., 2019), T follicular helper (TFH) cells, and activated NK Cells and M0 macrophages formed an immune risk score to be independent predictors for malignant transformation in low-grade glioma (Lu et al., 2019); higher percentage of CD163^+^ cells was associated with a worse prognosis in GBM (Martinez-Lage et al., 2019). This immune evaluation could help the design and implementation of effective therapeutic approaches in glioma patients. But the previous work neglected mast cells and neutrophils. We attempted to comprehensively analyze the immune cells that affect the individual’s immune determinants in order to more accurately assess the patient.

Various analytical methods had been developed to evaluate immune infiltration. The CIBERSORT (Cell type Identification By Estimating Relative Subsets Of Known RNA Transcripts) (Newman et al., 2015) algorithm was recently proposed for accurately quantifying the relative levels of leukocyte subsets with a high level of heterogeneity of gene expression within solid tumors, normal tissues, and blood in both healthy subjects and patients. In this study, CIBERSORT was used to estimate the proportions of 22 immune cell lineages. We additionally used least absolute shrinkage and selection operator (LASSO) as a method for regression approach with high-dimensional features, applying it to the Cox proportional hazard regression model for survival analysis. Finally, an immunoscore for predicting overall survival (OS) of patients with GBM in the training cohort was constructed using LASSO.

## Materials and Methods

### Collection and Preparation of Glioblastoma Datasets

**FIGURE 1:**
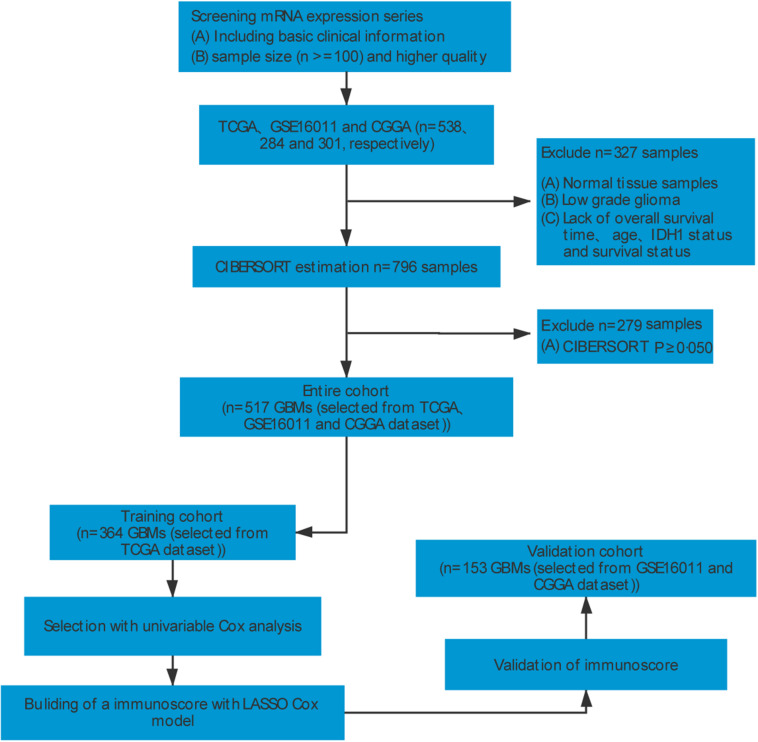
Flowchart illustrating data collection and analysis.

To identify GBM gene expression datasets with relevant clinical data, systematic computerized searches of Gene Expression Omnibus (GEO)^1^ datasets and The Cancer Genome Atlas^2^ were conducted. Our study design is shown in Figure 1. Inclusion and exclusion criteria for each dataset were applied in all cases: (a) normal control samples were excluded; (b) all datasets required necessary clinical information for age, IDH1 status, OS, and survival status; (c) all samples were pathologically diagnosed as GBM; (d) datasets with larger sample sizes (*n* ≥ 100) and higher quality were preferred. Finally, GBM datasets from TCGA (The Cancer Genome Atlas; United States)^3^ (Cancer Genome, 2008), GSE16011 (Netherlands)^4^ (Gravendeel et al., 2009), and CGGA (Chinese Glioma Genome Atlas; China)^5^ (Yan et al., 2012; Sun et al., 2014) were identified in this study. For Affymetrix expression arrays, the raw microarray data were downloaded and normalized using the affy package. For others, normalized matrices were downloaded directly. For genes with several probes, the median of all probes was chosen to represent its expression level.

### Estimation of Immune Cell Infiltration

To quantify the relative levels of distinct immune cells in GBM samples, CIBERSORT and a signature gene file, LM22, were used. LM22 consisted of 547 genes that accurately distinguish 22 mature human hematopoietic populations and activation states, including seven T-cell types, naïve and memory B cells, plasma cells, NK cells, and myeloid subsets. The abundances of immune cells of three datasets were estimated separately and the sum of all fractions was equal to 1 for a mixed sample. Furthermore, CIBERSORT also provided a P-value to filter out samples with non-significant data, providing a measure of statistical confidence. Only patients with a CIBERSORT *P*-value < 0.05 were considered eligible for further analysis.

### Grouping Method

After sample filtering, we grouped the TCGA cohort into a training cohort for identifying and evaluating predictors. Chinese Glioma Genome Atlas and GSE16011 datasets were used as validation cohorts to authenticate the immunoscore and nomogram.

### Statistical Analysis

Two groups were compared using the t-test for continuous variables. Hazard ratios for univariable analysis were calculated using a univariable Cox proportional hazards regression model. The LASSO Cox regression model was applied to determine the ideal coefficient for each prognostic feature and estimate the likelihood deviance (Tibshirani, 1997; Zhang and Lu, 2007), the equation of the used LASSO COX regression model as below. The optimal values of the penalty parameter λ were determined by 10-fold cross-validations. The coefficients and partial likelihood deviance were calculated with the R “glmnet” package^6^ (Friedman et al., 2010).

$$\underset{\beta_{0},\beta }{\text{min}}\frac{1}{N}\,\sum_{i=1}^{N}\omega_{i}\,l\,\left(y_{i},\beta_{0}+\beta^{T}\,x_{i}\right)+\lambda \,{||\beta ||}_{1}$$

Survival curves were constructed using the Kaplan–Meier method and means were compared by log-rank test. The optimal cut-off values were evaluated based on the association between OS and immune cell fraction in the training cohort using the “survminer” package.^7^ We investigated the prognostic or predictive accuracy of immunoscore using time-dependent receiver operating characteristic (ROC) analysis. The area under the curves at different cut-off times was used to measure prognostic or predictive accuracy, using the timeROC package.^8^ The Cox regression model was used to perform multivariable survival analysis and generate nomograms. Calibration curves were used to assess whether actual outcomes approximated predicted outcomes for nomogram. Nomogram and calibration curves were prepared with the rms package.^9^ The discrimination of the nomogram was calculated and compared by C-index. The methods used to compare the mean of two or more groups were presented in the ggpubr package. Gene set enrichment analysis (GSEA) was used to identify the pathways that were significantly enriched between High and Low immunoscore groups. All statistical analyses were performed using R (version 3.4.3).^10^ All statistical tests were two-sided and *P* < 0.05 was considered to indicate statistical significance.

## Results

### Construction of the Immunoscore

**FIGURE 2:**
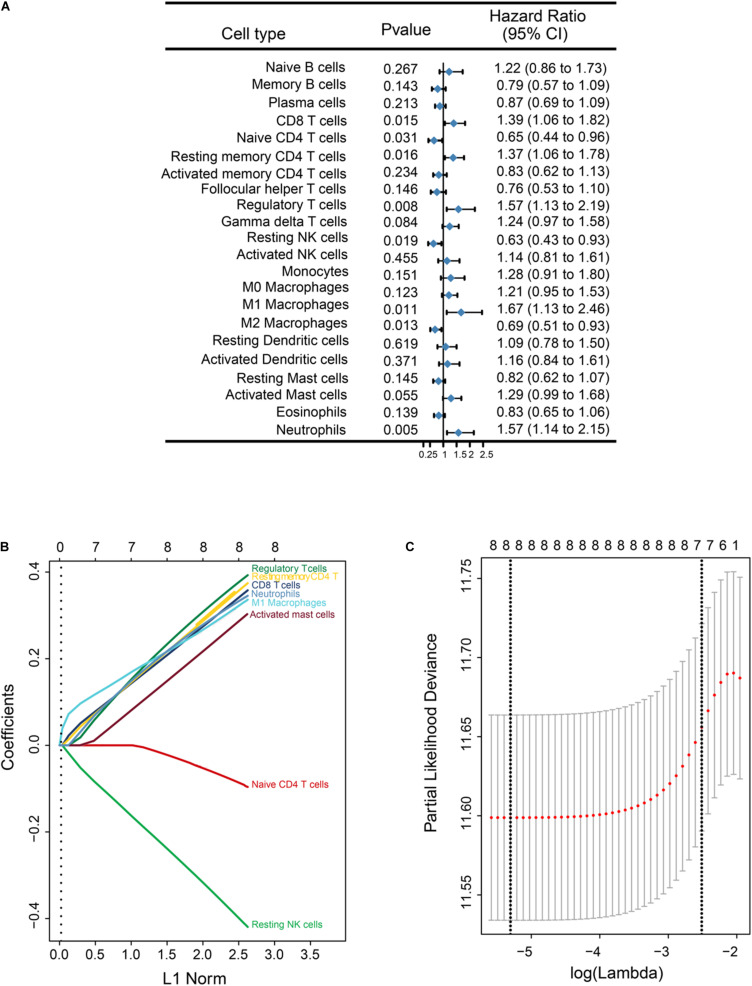
Overall survival (OS)-related immune cell infiltration in GBM samples, and corresponding LASSO analysis. **(A)** Forest plots showing associations between different immune cell types and OS in the training cohort. Hazard ratios are shown with 95% confidence intervals. **(B)** LASSO coefficient profiles of the eight OS-associated immune cells. The L1 norm is the regularization term for LASSO. Each curve corresponds to a cell type and represents the path of ico efficient against the L1-norm of the whole coefficient vector as λ varies. The corresponding cells are annotated at the end of the curve. A vertical line is drawn at the value chosen by 10-fold cross-validation. The upper x-axis represents the number of cell types involved in the LASSO model **(C)** Partial likelihood deviance for the LASSO coefficient profiles. The partial likelihood deviance is plotted against log (λ), where λ is the tuning parameter. Partial likelihood deviance values are shown on the y axis. The bold dashed vertical line shows the minimum criteria and the 1 standard error of the minimum criteria (the 1-SE criteria).

After data filtering and sorting using CIBERSORT, a total of 517 GBM samples with OS information were used for further analysis. A univariable COX analysis was used to calculate hazard ratios and *P*-values for each immune cell in the training cohort (*n* = 364) based on cut-off values (Supplementary Table 1). Unadjusted hazard ratios are shown with 95% confidence intervals. Partial immune cell subsets showed a significant association with OS (Figure 2A).

Given the strong outcome correlation, eight immune cell types in the training cohort were selected to construct an immunoscore using LASSO COX regression models. A vertical line was drawn at the value selected using 10-fold cross-validation. The coefficients for these eight features were calculated based on a minimized λ (Figure 2B). A coefficient profile plot was produced against the log(λ) sequence (Figure 2C). The partial likelihood deviance for minimized λ was 11.589, and the following formula was used: immunoscore = (0.321 × CD8 T cells) + (−0.075 × Naive CD4 T cells) + (0.336 × Memory resting CD4 T cells) + (0.357 × Regulatory T cells) + (−0.374 × Resting NK cells) + (0.305 × M1 Macrophages) + (0.264 × Activated Mast cells) + (0.317 × Neutrophils). In the formula, the level of one type of immune cell that was less than the corresponding cut-off value was equivalent to 0; otherwise, a value of 1 was assigned.

### Immunoscore Is Strongly Associated With OS

**FIGURE 3:**
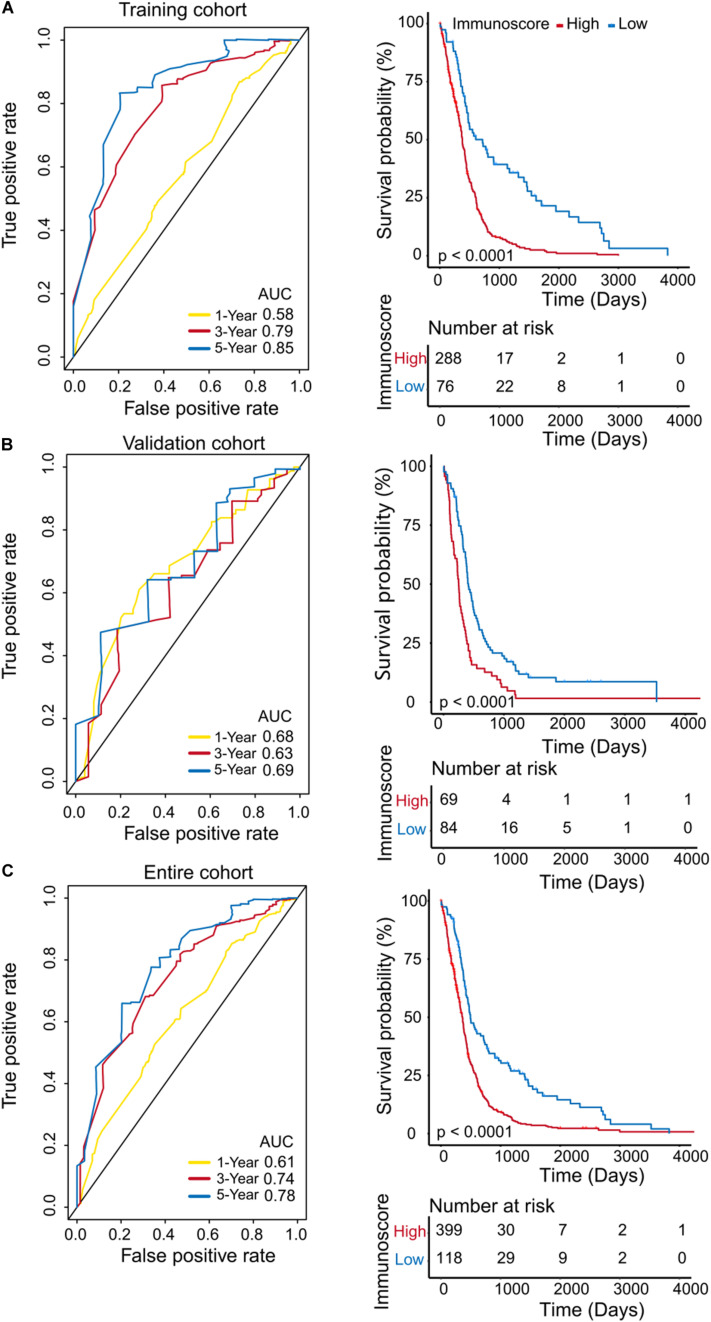
Evaluation of the prognostic accuracy of the immunoscore by time-dependent ROC analysis. Time-dependent ROC analysis was conducted at 1, 3, and 5 years, and Kaplan–Meier survival analysis was performed for patients in the High and Low immunoscore groups in the training cohort **(A)**, validation cohort **(B)**, and entire cohort **(C)**.

To evaluate the prognostic accuracy of immunoscore as a continuous variable, we conducted a time-dependent ROC analysis at 1, 3, and 5 years (Figure 3A). All patients in the training cohort were divided into High (*n* = 288) and Low immunoscore (*n* = 76) groups according to the cut-off point (0.850). The OS for patients in the Low immunoscore group was 73% at one year, 40% at three years, and 22% at five years, compared with 53, 7, and 2% in patients from the High immunoscore group, respectively, at these time points.

To confirm that the immunoscore model had similar prognostic value in different populations, the same formula was applied to the validation cohort and to the entire cohort. The prognostic accuracy of the immunoscore as a continuous variable in the validation and the entire cohorts was also assessed using time-dependent ROC analysis (Figures 3B,C). In both cohorts, patients were classified into Low and High immunoscore groups using the established cut-off point (validation cohort: 1.003, entire cohort: 0.886). The 1-, 3-, and 5-year OS rates of the Low immunoscore group were significantly higher than those of the High immunoscore group. After the model was adjusted for age (HR = 2.00; 95%CI = 1.61–2.49) and IDH1 status (HR = 0.48; 95%CI = 0.35–0.67) of samples, the multivariable Cox regression analysis also showed that immunoscore (HR = 2.43;95%CI = 1.86–3.19) was an independent prognostic indicator for OS (Table 1).

**TABLE 1:** Multivariate Cox analysis of clinicopathologic factors and immunoscore in entire cohort.

	**Multivariate cox**		
**Factor**	**HR**	**95%CI**	***P*-value**
Age	2.00	1.61–2.49	<0.001
Immunoscore	2.43	1.86–3.19	<0.001
IDH1 status	0.48	0.35–0.67	<0.001

### Immunoscore and Genetic Subgroups

**FIGURE 4:**
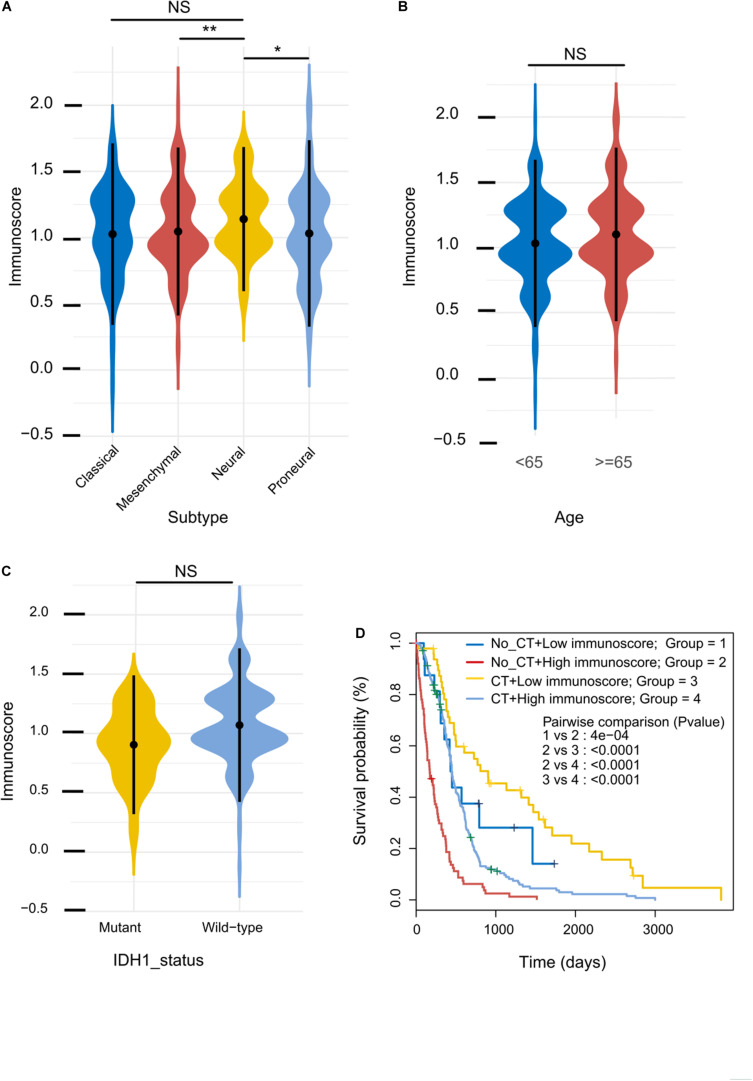
Immunoscore distribution and its correlation with sensitivity to chemotherapy. **(A–C)** Distribution of immunoscore in different subgroups in the training cohort. **(D)** Kaplan–Meier survival analysis between patients stratified by both adjuvant chemotherapy (CT) and immunoscore.

Based on the unique gene expression profiles, GBM can be stratified into four subgroups: mesenchymal, classical, proneural, and neural (Verhaak et al., 2010). We assessed the immunoscore as a continuous variable in these four subgroups, and we found that the immunoscore of the neural subgroup was significantly higher than that of the other subgroups in the training cohort (*P*-value: mesenchymal vs neural, 0.0079; proneural vs neural, 0.012; Figure 4A). However, the immunoscore distribution was independent of patients’ age and IDH1 status (Figures 4B,C). Furthermore, the mRNA levels of neuronal markers, such as NEFL, GABRA1, SYT1, and SLC12A5, were not significantly different between High and Low immunoscore groups (Supplementary Figure 1).

### Immunoscore and Adjuvant Chemotherapy

Adjuvant chemotherapy significantly enhanced OS in the Low immunoscore group (Figure 4D); by contrast, patients in the High immunoscore group who did not undergo adjuvant chemotherapy had a relatively poor prognosis. These results indicate that the immunoscore can successfully identify patients who are more likely to benefit from adjuvant chemotherapy.

### Construction of a Predictive Nomogram

**FIGURE 5:**
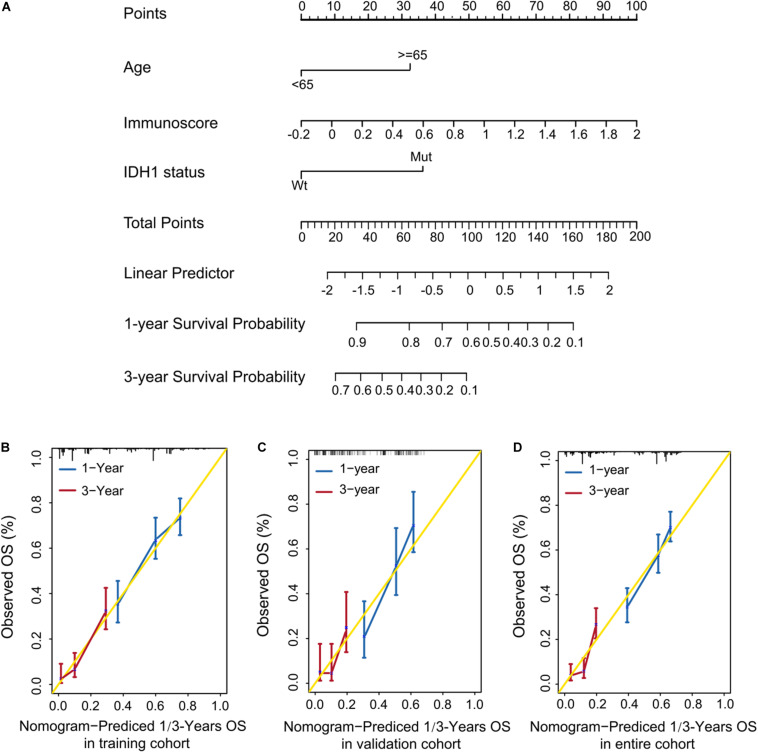
Quantitative prediction of OS in patients with GBM. **(A)** Nomogram to predict the 1- and 3-year OS. **(A–C)** Calibration curve for OS nomogram model in the training cohort **(B)**, validation cohort **(C)**, and entire cohort **(D)**.

To develop a quantitative method to predict patients’ OS, we constructed a nomogram using the training cohort. The predictors included age, immunoscore, and IDH1 status (Figure 5A). The calibration curves for one- and three-year OS were accurately predicted in the three cohorts (C-index: 0.66 for the training cohort, 0.67 for the validation cohort, and 0.68 for the entire cohort; Figures 5B–D). Our one-year decision curve analysis also showed that the nomogram had higher net benefit than IDH1 status across a range of risk thresholds in the training cohort. However, the net benefit for three-year OS was altered (Supplementary Figure 2).

### Identification of Immunoscore-Associated Biological Signaling Pathways and Immunogenic Molecules

**FIGURE 6:**
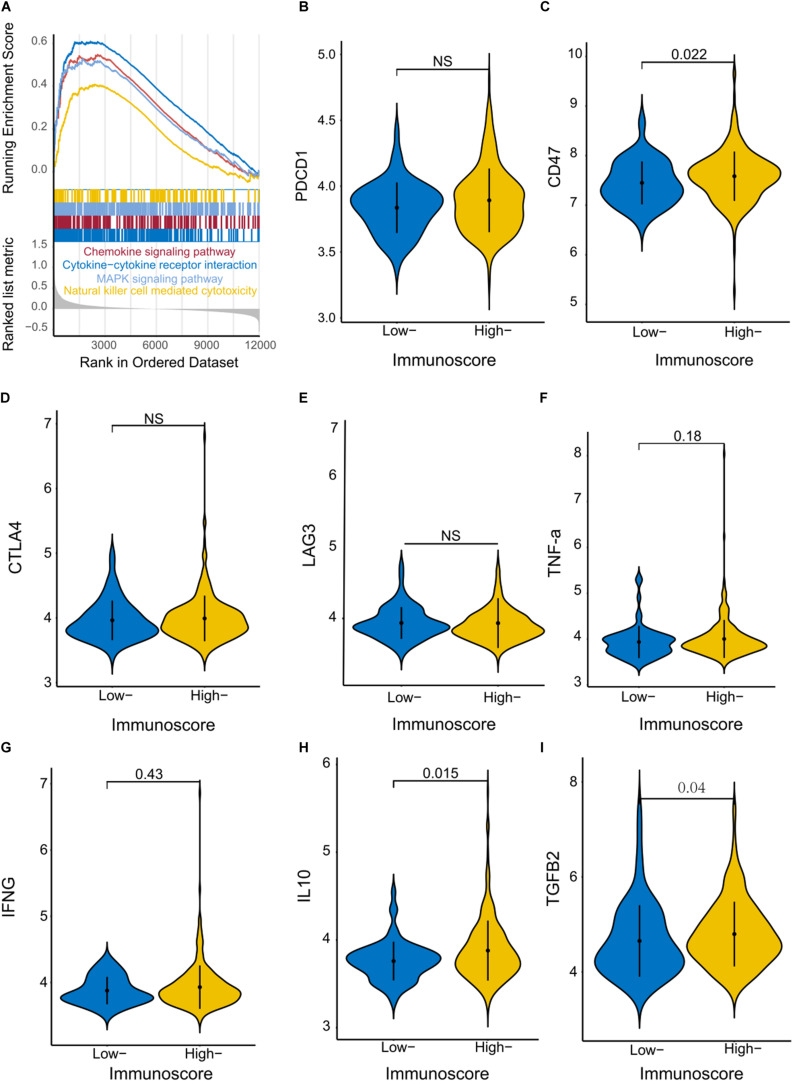
Identification of immunoscore-associated signaling pathways and differential expression of immune molecules. **(A)** The enriched biological pathways correlated with different immunoscore groups were identified with GSEA in the training cohort. Violin plots show the expression of immune checkpoint regulators **(B–E)** and inflammatory mediators **(F–I)** in the High and Low immunoscore groups.

We performed GSEA using the training cohort to identify immunoscore-associated biological signaling pathways. Significant gene sets (*P* value < 0.05) were visualized in an enrichment map (Figure 6A). The immunoscore was associated with several immune-related biological processes such as chemokine signaling, cytokine–cytokine receptor interaction, natural killer cell-mediated cytotoxicity, and MAPK signaling. Furthermore, we compared the levels of several immune checkpoint molecules between High and Low immunoscore groups in the training cohort. CD47 expression was significantly higher in the High immunoscore group (*P* = 0.022), while there were no significant differences in the levels of PDCD1 (programmed cell death 1; PD-1), CTLA4, and LAG expression (Figures 6B–E). The expression of two cytokines (IL-10 and TGF-β2) was significantly higher in the High immunoscore group (Figures 6F–I).

## Discussion

GBM exhibits a remarkable level of heterogeneity; therefore, the integration of multiple biomarkers into a single model has the potential to offer improved prognostic value over the use of each biomarker individually. The correlation between tumor-infiltrating immune cells and patient outcome has been well documented in melanoma (Tefany et al., 1991; Clemente et al., 1996) as well as ovarian (Zhang et al., 2003; Sato et al., 2005; Hamanishi et al., 2007), head and neck (Shibuya et al., 2002; Badoual et al., 2006), bladder (Nakakubo et al., 2003), breast (Mahmoud et al., 2011), urothelial (Sharma et al., 2007), colorectal (Pages et al., 2005; Mlecnik et al., 2011), renal (Nakano et al., 2001), prostatic (Karja et al., 2005), gastric (Jiang et al., 2018), and lung cancer (Al-Shibli et al., 2008; Dieu-Nosjean et al., 2008). However, compared with these tumors, GBM has a lower tumor mutation burden and a higher immunosuppressive tumor microenvironment. PD-L1 is one of the molecular mechanisms of immunosuppression. There are some studies about PD-1 inhibitors (nivolumab or pembrolizumab) in treatment of GBM. Chimeric antigen receptor (CAR) T cell therapy is another part of immunotherapeutic approaches. Importantly, CAR T cells can penetrate the blood brain barrier. Currently, several related clinical trials have demonstrated the safety of CAR T cells in central nervous system tumor (Zhao et al., 2019). The above studies show that immunotherapy and immune microenvironment play a more important role in the treatment of glioma. In the present study, the immunoscore was developed and validated based on the fractions of eight selected immune cell types and was used as a novel prognostic tool independent of other clinical characteristics. Our results show that this tool can be successfully used to categorize patients into High and Low immunoscore groups, each showing markedly different OS. Infiltration of CD8 T cells, memory resting CD4 T cells, regulatory T cells, M1 macrophages, activated mast cells, and neutrophils indicated poor prognosis, whereas infiltration of naive CD4 T cells and resting NK cells predicted favorable prognosis in patients with GBM. In human gliomas, macrophages are the primary immune subset. However, they do not appear to be fully able to modulate normal immune responses and are reported to exhibit weak tumoricidal activity and capacity to activate antitumor effector T cells (Hussain et al., 2006). Furthermore, hypoxia-induced tumor-associated macrophages are reported to be associated with poor prognosis, angiogenesis, and tumor invasiveness (Allavena et al., 2008). The cytokine profile of macrophages is similar to that of resting microglia, which suggests that tumor cell antigens do not activate macrophages within malignant gliomas. Macrophages also express the macrophage migration inhibitory factor (MIF), which contributes to immune escape and tumor progression (Mittelbronn et al., 2011). Functionally, regulatory T cells are reported to prevent the activation or expansion of both CD4 and CD8 T cells as well as suppressive cytokines (IL-10, TGF-β) that are involved in restricting antigen-presenting capacity and cell-to-cell contact inhibition (Ooi et al., 2014). Although the significance of regulatory T cells in patients with malignant glioma is yet to be determined, their presence has been found to be a negative prognostic indicator in patients with ovarian carcinoma. Current studies demonstrate that regulatory T cells within malignant gliomas also suppress pro-inflammatory cytokine production (TNF-a, IFN-γ, and IL-6) and impair antigen presentation by monocytes and macrophages in a process that is distinct from their inhibition of effector CD4 and CD8 T-cell activity. Furthermore, the expression of genes involved in TCR antigen binding, activation, and intracellular signaling function is notably decreased. Conversely, the expression of genes involved in inhibiting the immune response was previously found to be significantly upregulated in regulatory T cells (Learn et al., 2006). Thus, in glioma patients, regulatory T cells appear to play a role in immune escape by modulating immune responses (Hussain et al., 2006); therefore, immunotherapy against regulatory T cells may represent a potential therapeutic approach.

There is also evidence that the proinflammatory activities of mast cells sustain the tumor microenvironment. In particular, mast cells play a role in tumor initiation, maintenance, and growth by synthesizing and secreting matrix metalloproteinases, various cytokines (IL6 and TNFα), and multiple mitogens [vascular endothelial growth factor precursor (VEGF) and platelet-derived growth factor (PDGF)] (Galli and Tsai, 2008; Galli et al., 2008). In glioma, the accumulation of mast cells is more pronounced in patients with higher grades of malignancy, indicating that their presence is detrimental. This correlation suggests that gliomas secrete factors that recruit mast cells, which may promote glioma development (Polajeva et al., 2014). Macrophages and neutrophils are enriched in glioma and indicate poor prognosis (Zhang et al., 2017). Notably, our results demonstrate that the levels of suppressive cytokines (IL-10 and TGF-β) were increased along with the expression levels of CD47 in the High immunoscore group. This finding may indicate that CD47 plays an important role in the response to immunotherapy in patients with GBM. Immunoscore can be used as an independent prognostic factor; the patients with high immunoscore have worse outcome. Meanwhile, the combination of the immunoscore with assessment of traditional risk factors (age and IDH1-status) resulted in a potentially useful tool for the prediction of GBM prognosis. The immunoscore, as part of diagnostic and prognostic assessment of GBM, may provide crucial novel prognostic information, facilitating clinical decision making, including the rational stratification of patient treatment strategies. For patients newly diagnosed with GBM, current standard treatment includes maximal surgical resection followed by combined radiotherapy plus concomitant and maintenance chemotherapy with temozolomide (TMZ). As the only indicator of chemosensitivity, methylation of the MGMT promoter has been shown to predict response to alkylating agents. Its status may play a crucial role in the choice of single modality treatment in patients with GBM (Hegi et al., 2005; Stupp et al., 2005). Previous studies demonstrate that immune infiltration is associated with sensitivity to chemotherapy in several types of cancers (Zitvogel et al., 2008; Galluzzi et al., 2015; Jiang et al., 2018). Consistent with these findings, our studies underscore the importance of identifying Low immunoscore patients who are more likely to benefit from adjuvant chemotherapy. However, the mechanisms underlying this phenomenon are not yet clear.

This study had several limitations. First, it was a retrospective study based on publicly available data sets, and it was not possible to obtain all the information required to confirm our hypothesis. We may have also neglected other important prognostic and predictive markers in our study. Second, the cut-off value for the immunoscore needs further improvement. Third, it was unclear how the presence of these immune cells affected patient prognosis and therapeutic outcome. Finally, we do not adjust molecular subgroup, sample size, mutation burden, PD-L1 expression, and others. Therefore, the immunoscore may be an independent prognostic factor, and further studies are required to characterize the clinical use of immunoscore in predicting the prognosis of GBM. In summary, our findings show that the immunoscore can be effectively applied to classify patients with GBM into High and Low immunoscore groups, thereby adding prognostic value to the traditional risk factors used to assess their prognosis. Moreover, the immunoscore may represent potential predictive indicator to identify patients who would benefit from adjuvant chemotherapy. Thus, the immunoscore potentially offers clinical value in prescribing a personalized therapeutic regimen.

## Data Availability Statement

Publicly available datasets were analyzed in this study. This data can be found here: https://www.ncbi.nlm.nih.gov/geo/, https://portal.gdc.cancer.gov/, https://gdc.cancer.gov/, http://www.ncbi.nlm.nih.gov/geo/query/acc.cgi?acc=GSE16011, and http://www.cgga.org.cn.

## Author Contributions

XT and QC designed the research. PX, AC, GD, SZ, and LG collected and analyzed the data. XT and LD wrote and revised the manuscript. All authors contributed to the article and approved the submitted version.

## Conflict of Interest

The authors declare that the research was conducted in the absence of any commercial or financial relationships that could be construed as a potential conflict of interest.

## Footnotes

1 https://www.ncbi.nlm.nih.gov/geo/

2 http://cancergenome.nih.gov/dataportal/

3 https://gdc.cancer.gov/

4 http://www.ncbi.nlm.nih.gov/geo/query/acc.cgi?acc=GSE16011

5 http://www.cgga.org.cn

6 https://CRAN.R-project.org/package=glmnet

7 https://CRAN.R-project.org/package=survminer

8 https://CRAN.R-project.org/package=timeROC

9 https://CRAN.R-project.org/package=rms

10 www.r-project.org

## Abbreviations

LASSO: least absolute shrinkage and selection operator
GBM: glioblastoma multiform
EGFR: epidermal growth factor receptor
IDH1/2: isocitrate dehydrogenase
MGMT: O6-methylguanine-DNA methyltransferase
OS: overall survival
GEO: Gene Expression Omnibus
TCGA: The Cancer Genome Atlas
CGGA: Chinese Glioma Genome Atlas
LM22: 22 immune cell types
ROC: receiver operating characteristic
GSEA: gene set enrichment analysis
NEFL: neurofilament light
GABRA1: gamma-aminobutyric acid type A receptor alpha1 subunit
SYT1: synaptotagmin 1
SLC12A5: solute carrier family 12 member 5
TMZ: temozolomide
IL: interleukin
TNF: tumor necrosis factor
TGF: transforming growth factor
VEGF: vascular endothelial growth factor precursor
PDGF: platelet-derived growth factor
PD-1: programmed cell death 1
CTLA4: cytotoxic T-lymphocyte associated protein 4
LAG3: lymphocyte activating 3
CT: chemotherapy
C-index: concordance index.
