# Supplementary figures and images for: Prognostic and Predictive Value of an Immunoscore Signature in Glioblastoma Multiform
Source: Front Genet. 2020 Nov 9;11:514363. doi: 10.3389/fgene.2020.514363 (PMC7684008; doi:10.3389/fgene.2020.514363)

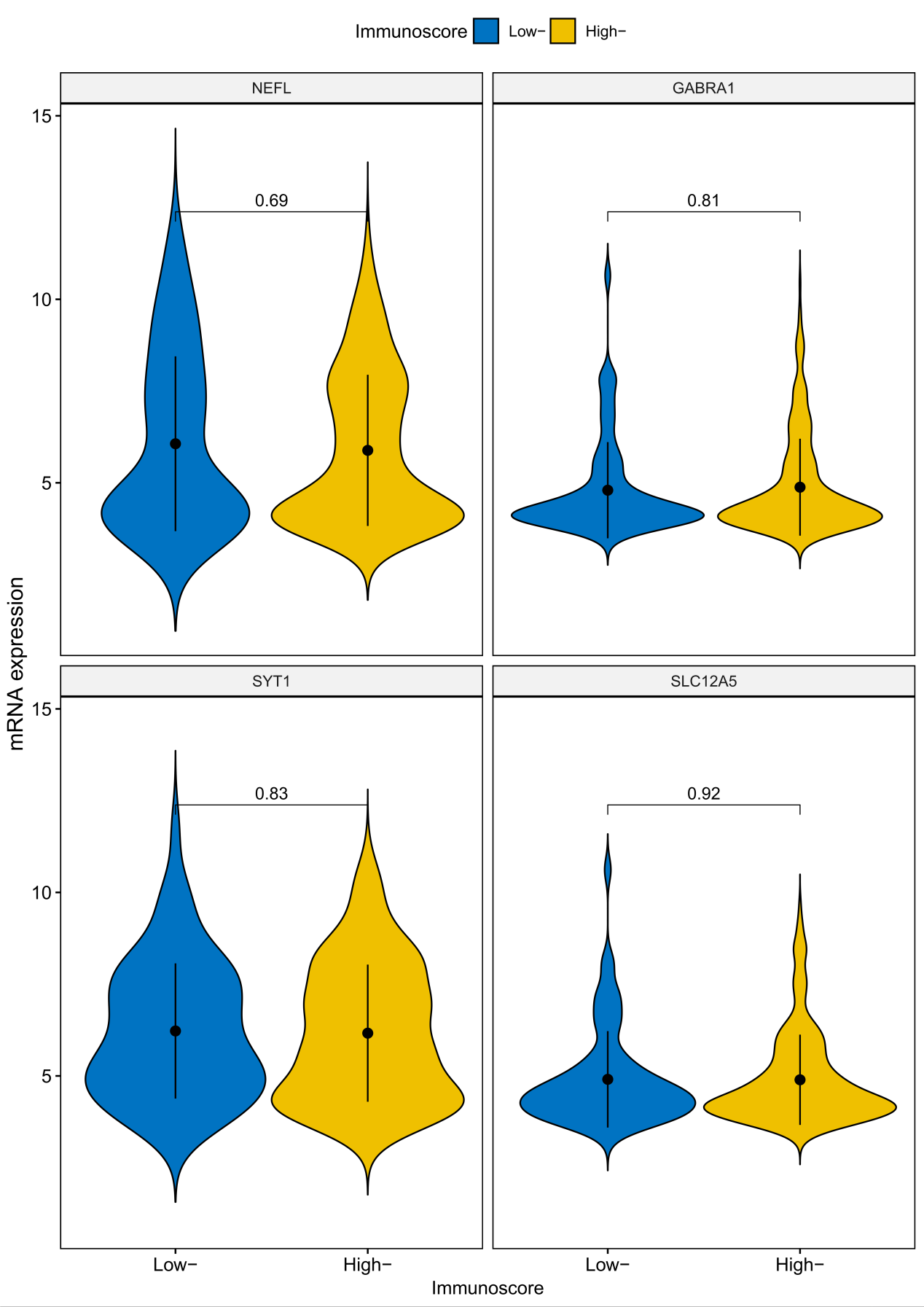

Supplement: Supplementary file 1 [file Image_1.tif]
