# Supplementary material for: Prognostic and Predictive Value of an Immunoscore Signature in Glioblastoma Multiform
Source: Front Genet. 2020 Nov 9;11:514363. doi: 10.3389/fgene.2020.514363 (PMC7684008; doi:10.3389/fgene.2020.514363)
Supplement: Supplementary file 2 [file Table_1.docx]

**Table S1** Cut-off value for 22 immune cell fractions

| Cell types Cutpoint |
| --- |
| B.cells.naive 0.019413980  B.cells.memory 0.045314757  Plasma.cells 0.002526518  T.cells.CD4.naive 0.029194683  T.cells.CD4.memory.activated 0.018860024  T.cells.follicular.helper 0.060434247  T.cells.gamma.delta 0.026010136  NK.cells.resting 0.027095998  NK.cells.activated 0.070333767  Monocytes 0.224807994  Macrophages.M0 0.320145719  Dendritic.cells.resting 0.004305207  Dendritic.cells.activated 0.016760526  Mast.cells.resting 0.019946606  Mast.cells.activated 0.000100000  Eosinophils 0.001329062  T.cells.CD8 0.006437244  T.cells.CD4.memory.resting 0.004319686  T.cells.regulatory.Tregs 0.021356811  Macrophages.M1 0.003550880  Macrophages.M2 0.171584924  Neutrophils 0.091048952 |
